# Differential cytokine regulation by NF-κB and AP-1 in Jurkat T-cells

**DOI:** 10.1186/1471-2172-11-26

**Published:** 2010-05-27

**Authors:** Hazem Khalaf, Jana Jass, Per-Erik Olsson

**Affiliations:** 1 Biology, Örebro Life Science Center, School of Science and Technology, Örebro University, SE-701 82 Örebro, Sweden; 2 The Lawson Health Research Institute and Department of Microbiology and Immunology, University of Western Ontario, London, Ontario, N6A 4V2 Canada

## Abstract

**Background:**

Activator protein (AP)-1 and nuclear factor (NF)-κB largely control T-cell activation, following binding of foreign antigens to the T-cell receptor leading to cytokine secretion. Elevated levels of pro-inflammatory cytokines and chemokines such as TNF, IL-6 and CXCL8 are associated with several human diseases including cystic fibrosis, pulmonary fibrosis and AIDS. The aim of this study was to investigate the role of the transcription factors, AP-1 and NF-κB, in IL-6 and CXCL8 regulation in Jurkat T-cells.

**Results:**

Phorbol myristate acetate (PMA) exposure resulted in an up-regulation of AP-1 and down-regulation of NF-κB activity, however, exposure to heat killed (HK) *Escherichia. coli *MG1655 resulted in a dose-dependent increase in NF-κB activity without affecting AP-1. The cytokine profile revealed an up-regulation of the chemokine CXCL8 and the pro-inflammatory cytokines TNF, IL-2 and IL-6 following treatment with both PMA and HK *E. coli*, while the levels of the anti-inflammatory cytokine IL-10 were not affected by PMA but were significantly down-regulated by HK *E. coli*. AP-1 activation was significantly increased 2 h after PMA exposure and continued to increase thereafter. In contrast, NF-κB responded to PMA exposure by a rapid up-regulation followed by a subsequent down-regulation. Increased intracellular Ca^2+ ^concentrations countered the down-regulation of NF-κB by PMA, while similar treatment with calcium ionophore resulted in a reduced NF-κB activity following induction with HK *E. coli*. In order to further study NF-κB activation, we considered two up-stream signalling proteins, PKC and Bcl10. Phosphorylated-PKC levels increased in response to PMA and HK *E. coli*, while Bcl10 levels significantly decreased following PMA treatment. Using an NF-κB activation inhibitor, we observed complete inhibition of IL-6 expression while CXCL8 levels only decreased by 40% at the highest concentration. Treatment of Jurkat T-cells with PMA in the presence of JNK-inhibitor suppressed both CXCL8 and IL-6 while PKC-inhibitor primarily decreased CXCL8 expression.

**Conclusion:**

The present study shows that NF-κB regulated IL-6 but not CXCL8. This complex regulation of CXCL8 suggests that there is a need to further evaluate the signalling pathways in order to develop new treatment for diseases with elevated CXCL8 levels, such as AIDS and autoimmune diseases.

## Background

Cytokines and chemokines are important in immune cell recruitment and in regulation of inflammatory responses [1]. T-cells produce a broad range of inflammatory mediators, including IL-2, IL-6, TNF and CXCL8, all of which are important in cell proliferation, differentiation, communication and initiation of inflammatory responses [2]. Elevated levels of pro-inflammatory cytokines and chemokines, such as TNF, IL-6 and CXCL8, are associated with several human diseases including cystic fibrosis [3-5], pulmonary fibrosis [6,7] and AIDS [8,9]. Induction of CXCL8 has been suggested to be mediated through NF-κB in cooperation with AP-1 [10,11], however the precise mechanism is not fully elucidated, and treatment strategies aimed at inhibiting CXCL8 have failed [12]. Persistent production of IL-6 and CXCL8 leads to chronic inflammation and enhanced survival of lymphocytes increasing serum cytokine/chemokine levels. This forms the basis of several autoimmune disorders including plasmacytosis and hyperplasia [13]. To develop viable CXCL8 based treatment strategies, it is necessary to identify the signalling pathways regulating CXCL8 and determine how this is coupled to NF-κB, AP-1 and IL-6.

The signalling pathways leading to NF-κB and AP-1 activation are overlapping, where both are involved in the induction and regulation of cytokines/chemokines. NF-κB is activated in response to stress, such as oxidative stress, bacterial toxins, viruses and UV light [14], and is essential for differentiation, proliferation and survival of many cell types including T-lymphocytes [15]. AP-1 activation requires Fos (c-Fos, FosB, Fra-1, Fra-2) and Jun (c-Jun, v-Jun, JunB, JunD) through the formation of homo- and hetero-dimers [16,17], and regulates transcription of a broad range of genes involved in immune responses [18-21]. Both AP-1 and NF-κB binding sites have been identified in the promoter region of IL-6 and CXCL8 [12,22], however, the mechanism by which these interleukins are regulated in T-cells is still not clear. CXCL8 is a C-X-C chemokine with properties enabling it to recruit T-cells and basophils and to activate neutrophils and monocytes [23]. IL-6 is a cytokine that possesses both pro- and anti-inflammatory characteristics and that plays a key role in haematopoiesis and acute-phase responses [24,25].

The present study suggests that the regulation of CXCL8 and IL-6 is uncoupled. Using Jurkat T-cells exposed to PMA and heat killed (HK) *Escherichia coli *MG1655 in combination with inhibitors of NF-κB, JNK and PKC, we demonstrated that NF-κB regulates IL-6 expression while the regulation of CXCL8 more closely correlated to AP-1 activity. These results indicate that inhibition of NF-κB is not an effective strategy in countering the high CXCL8 activities in diseases such as cystic fibrosis, AIDS and pulmonary fibrosis.

## Results

### Regulation of AP-1 and NF-κB activation

**Figure 1:**
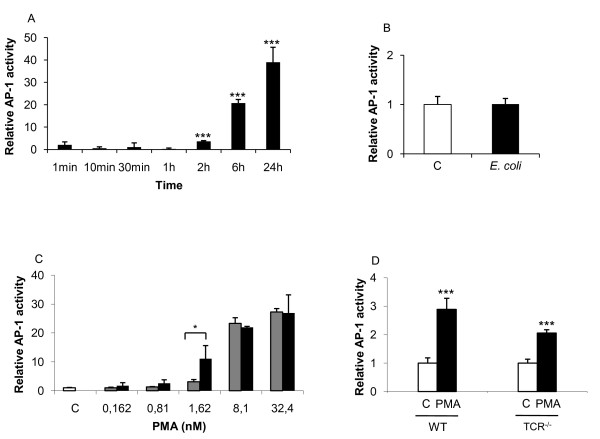
**AP-1 activation following long-term exposure of Jurkat T-cells to PMA**. Jurkat T-cells were transfected with luciferase reporter plasmids containing the AP-1 cis-elements. **(A) **Time-dependent AP-1 activation in response to PMA. **(B) **HK *E. coli *does not activate AP-1. **(C) **Dose-dependent activation of AP-1 were performed using PMA alone (grey bars) and in combination with calcium ionophore (CaI 955 μM, black bars). (**D**) TCR^-/- ^deficient cells responded to PMA (162 nM) by up-regulating AP-1 activity. Statistical significance from the control was determined using Student's *t*-test. (n = 4). Controls were arbitrarily set to 1.

The transcription factors NF-κB and AP-1 play key roles in the initiation of an inflammatory response by inducing the expression and secretion of chemokines and cytokines that attract and activate immune cells. However, the signal transduction pathways and subsequent inflammatory cytokine induction by these transcription factors is not fully elucidated. The present study is aimed at determining the involvement of AP-1 and NF-κB in cytokine induction and regulation. PMA treatment resulted in an up-regulation of AP-1 after 2 h exposure and continued to increase throughout the analysis period (figure 1a). HK *E. coli *treatment did not affect AP-1 activation in Jurkat T-cells (figure 1b). To determine the involvement of associated pathways, we exposed cells to Ca^2+ ^ionophore with or without PMA and observed a modest involvement of Ca^2+ ^in PMA-dependent AP-1 activation (figure 1c) while Ca^2+ ^alone did not alter AP-1 activity (data not shown). Furthermore, AP-1 activity decreased in a TCR-deficient Jurkat cell line when exposed to PMA compared to the parent cell line indicating that regulation of AP-1 was only partially T-cell receptor dependent (figure 1d).

**Figure 2:**
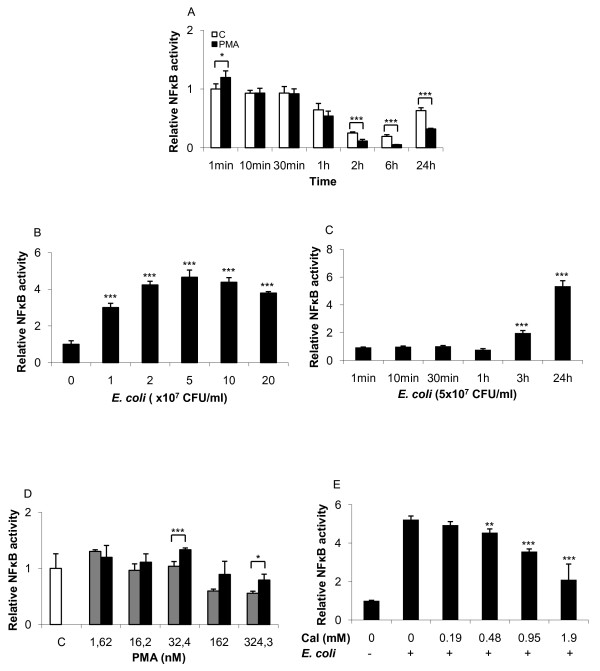
**NF-κB down-regulation by PMA and up-regulation by heat killed *E. coli *MG1655 following long-term stimulation**. Transfection of Jurkat T-cells was performed using luciferase reporter plasmids containing NF-κB cis-elements. (**A**) Time-dependent stimulation of Jurkat T-cells using PMA. NF-κB activation was evaluated using HK *E. coli *in a (**B**) dose- and (**C**) time-dependant manner. Calcium ionophore increased NF-κB activity following PMA exposure (**E**) and resulted in a negative regulation in response to HK *E. coli *stimulation (**D**). Statistical significance from the control was determined using Student's *t*-test. (n = 4). Controls were arbitrarily set to 1.

NF-κB levels showed a transient increase at 1 min after exposure to PMA (figure 2a). However, 1 h after exposure the NF-κB levels began to drop reaching the lowest levels by 6 h, after which they increased again by 24 h. Exposure of Jurkat T-cells to HK *E. coli *resulted in a dose-dependent NF-κB activation, with the highest activity observed at a relative concentration of 5 × 10^7 ^CFU/ml (figure 2b). The time-dependent activation of NF-κB by HK *E. coli *was assessed further using the optimal concentration obtained from figure 2b and showed that the NF-κB activity increased after 3 h of exposure (figure 2c). Furthermore, increased intracellular Ca^2+ ^reversed the PMA dependent NF-κB inhibition (figure 2d) and reduced the HK *E. coli *-dependent NF-κB activation (figure 2e).

### Induction of inflammatory responses

**Figure 3:**
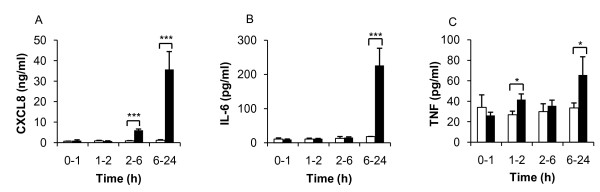
**CXCL8, IL-6 and TNF expression following long-term stimulation with PMA**. Jurkat T-cells were either treated with media (white bars) or stimulated with PMA (black bars) and incubated for 1 h, 2 h, 6 h and 24 h. Aged media was added following each centrifugation step representative to the stimulation time (see Materials and methods). Cytokine levels (**A**) CXCL8, (**B**) IL-6 and (**C**) TNF were detected by ELISA. Statistical significance from the control was determined using Student's *t*-test. (n = 3).

The ability of PMA and HK *E. coli *to induce an inflammatory response in Jurkat T-cells was evaluated using a multiplex cytokine assay following 24 h stimulation. The cytokine profile revealed an enhanced induction of the pro-inflammatory cytokines IL-2, IL-6, TNF and the chemokine CXCL8. The levels of the anti-inflammatory cytokine IL-10 were unaffected by PMA but were significantly decreased by HK *E. coli *(Table 1). These results confirmed that PMA and HK *E. coli *induced an inflammatory response in the Jurkat T-cells. It is interesting to note that PMA was 120-fold more effective at inducing CXCL8 than HK *E. coli*. PMA-dependent induction of AP-1 and down-regulation of NF-κB suggests an involvement of AP-1 in CXCL8 regulation. Determination of the time course of cytokine induction in response to PMA showed that CXCL8 was already released between 2-6 h, while TNF and IL-6 were released between 6-24 h (figure 3). These results indicated that the cytokines were differentially regulated and that the release was not associated with the early transient induction of NF-κB. The temporal induction of AP-1 correlated to the CXCL8 levels and preceded the TNF and IL-6 release. This suggests an association between CXCL8 release and AP-1 signalling.

**Table 1:** Jurkat T-cells were stimulated with 162 nM PMA and 5 × 10^7 ^CFU/ml HK *E. coli *for 24 h.

	C	HK *E. coli*	PMA
**IL-2**	0.25 ± 0.27	0.95 ± 0.06**	1.75 ± 0.16***
**IL-6**	6.65 ± 0.2	27.275 ± 1.47***	50.33 ± 2.64***
**CXCL8**	38.45 ± 5.02	253.08 ± 13.42***	≥30,000***
**IL-10**	13.07 ± 0.64	4.10 ± 0.11***	12.99 ± 0.92
**TNF**	1.21 ± 0.17	1.79 ± 0.26**	10.28 ± 0.65***

Cytokine levels were determined using multiplex assays. Concentrations are given as pg/ml for each group (mean value ± SD, n = 3). Statistical significance from the control (C) was determined using Student's *t*-test.

### Cooperative induction of cytokines by AP-1 and NF-κB

**Figure 4:**
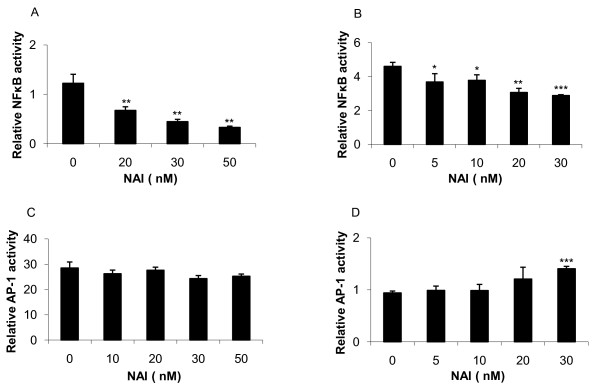
**Inhibition of NF-κB activity by NAI**. Jurkat T-cells were transfected with luciferase reporter plasmids containing either NF-κB or AP-1 cis-acting elements. The cells were incubated with NF-κB activation inhibitor (NAI) for 1 h followed by stimulation with PMA (162 nM, **A **and **C**) or HK *E. coli *(5 × 10^7 ^CFU/ml, **B **and **D**) for 24 h. **(A, B) **NF-κB, but not **(C, D) **AP-1 was inhibited. Statistical significance from the positive control (PMA/HK *E. coli*) was determined using Student's *t*-test. (n = 4). Controls were set to 1.

**Figure 5:**
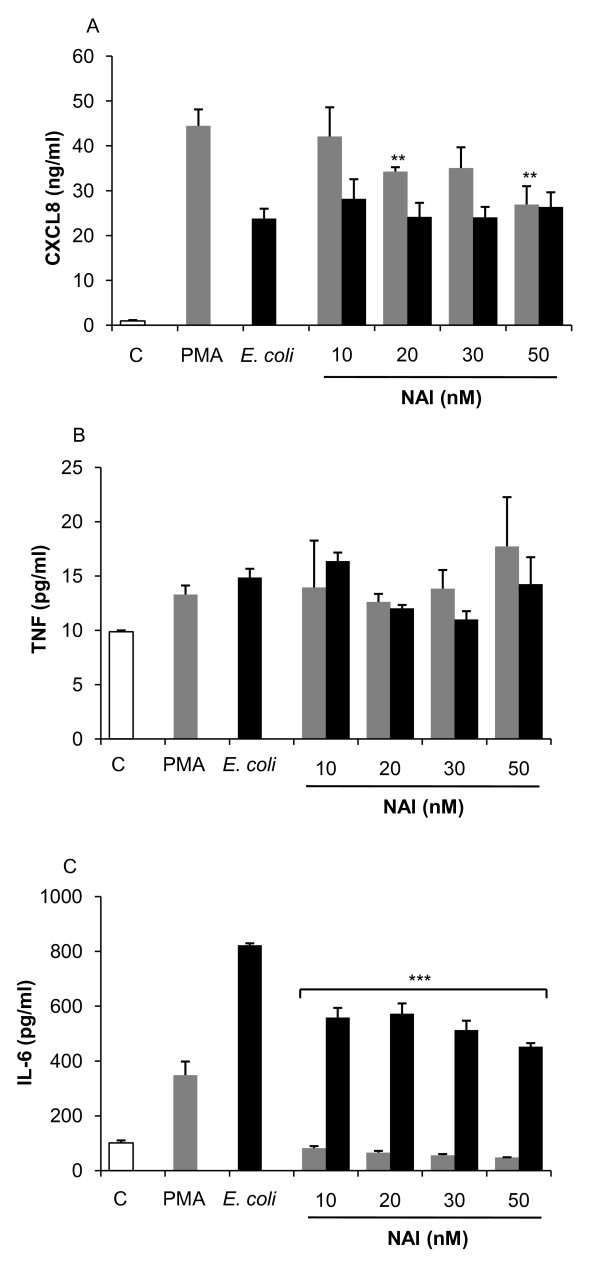
**Involvement of NF-κB in cytokine regulation**. Cytokine/chemokine levels were determined using ELISA following incubation of Jurkat T-cells with NAI (1 h) and stimulation (24 h). **(A) **CXCL8 expression was partially inhibited following PMA stimulation (grey bars), whereas the levels were not altered following stimulation with HK *E. coli *(black bars), this indicates an induction mainly regulated by AP-1. **(B) **TNF expression was not affected by NAI. **(C) **IL-6 release was completely inhibited by NAI following PMA exposure, indicating regulation through NF-κB since IL-6 expression was significantly increased in response to HK *E. coli *than PMA. Statistical significance from the positive control (PMA/HK *E. coli*) was determined using Student's *t*-test. (n = 3).

To further characterize the involvement of NF-κB in cytokine regulation, we treated cells with an NF-κB activation inhibitor (NAI). The results showed that NAI selectively down-regulated NF-κB activation (figures 4a and 4b) and did not alter AP-1 activity (figures 4c and 4d). Exposure of Jurkat T-cells to NAI resulted in a modest reduction of CXCL8 following PMA exposure, while it did not alter the CXCL8 release following HK *E. coli *exposure (figure 5a). NAI did not affect TNF expression (figure 5b) indicating that NF-κB is not the main regulator of CXCL8 or TNF following either PMA or HK *E. coli *exposure in Jurkat T-cells. In contrast, NAI resulted in a complete inhibition of IL-6 following PMA exposure and a 45% inhibition following HK *E. coli *exposure (figure 5c), suggesting an involvement of NF-κB in IL-6 regulation.

**Figure 6:**
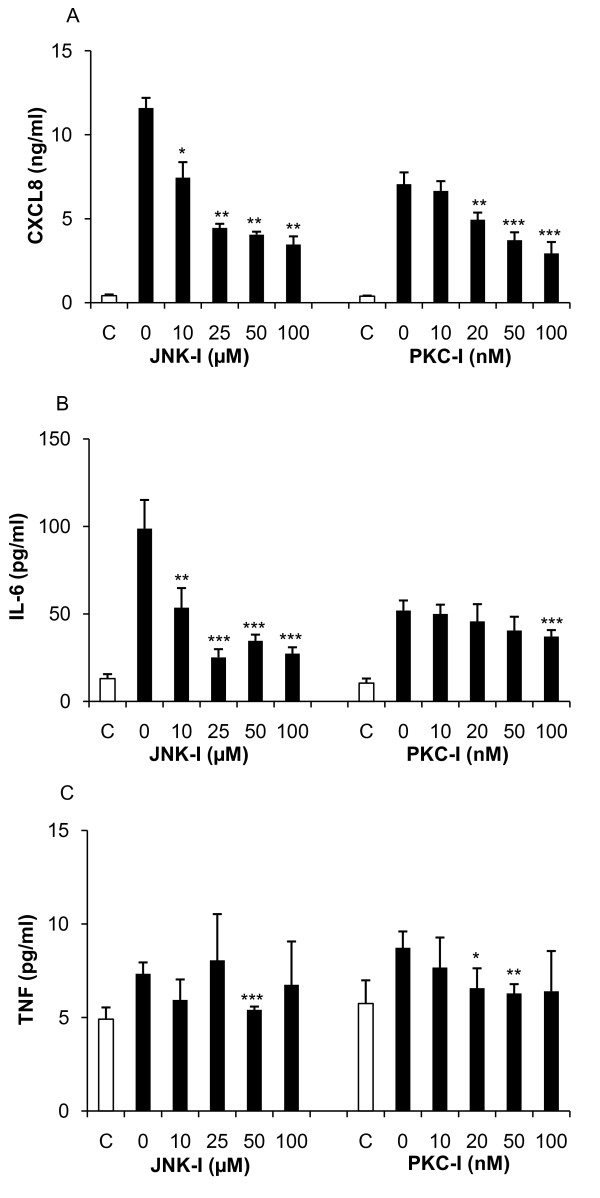
**Involvement of PKC and JNK in cytokine expression**. Jurkat T-cells were incubated with PKC- and JNK inhibitors for 1 h followed by stimulation with PMA (162 nM) for 24 h. **(A) **CXCL8 expression was inhibited in a dose-dependent manner by both inhibitors. **(B) **JNK-I revealed a stronger inhibitory effect on IL-6 expression than the PKC-I. **(C) **TNF expression was not affected by either inhibitor. Statistical significance from the positive control (PMA) was determined using Student's *t*-test. (n = 3).

Ca^2+ ^was observed to increase AP-1 activity (figure 1c) and reduce NF-κB activity (figure 2e); therefore, we exposed T-cells to a PKC inhibitor together with PMA to determine its effect on cytokine expression. Inhibition of PKC reduced CXCL8 release from 7 ng/ml to 3 ng/ml while it had a modest effect on IL-6 and TNF (figure 6a-c). This prompted us to test the effect of JNK inhibition on PMA-induced cytokine expression. JNK is involved in the regulation of a multitude of different transcription factors, including the phosphorylation and activation of c-Jun, c-Fos and p53, leading to cellular apoptosis [26]. Inhibition of the JNK pathways resulted in a down-regulation of both CXCL8 and IL-6, while no clear effect was observed on TNF expression (figure 6a-c). Analysis of mRNA levels using RT-qPCR (table 2) showed that PMA induced both *il-6 *and *cxcl8 *mRNA (5.1-fold and 111.8 fold respectively). Addition of the NF-κB inhibitor NAI and the JNK inhibitor reduced the *il-6 *expression below basal levels. In contrast, while the *cxcl8 *levels were suppressed by the same treatments the levels remained elevated above basal level.

**Table 2:** Jurkat T-cells were treated with 10 nM NAI, 10 μM JNK-I or10 nM PKC-I for 2 h followed by induction with 162 nM PMA for 24 h.

	C	PMA	NAI	JNK-I	PKC-I
**IL-6**	11.7 ± 2.4	63.6 ± 11.9	3.3 ± 4.0*	2.7 ± 2.2**	39.0 ± 20.8
**CXCL8**	450 ± 139	50294 ± 19161	7411 ± 545**	3197 ± 1079*	23238 ± 2547

Gene expression was determined using RT-qPCR. The Ct values were normalized against 18S. Statistical significance from the positive control (PMA) was determined using Student's *t*-test. Concentrations are given as fg/ml, values are presented as mean ± SEM, n = 3.

### NF-κB inhibition due to PKC-dependent Bcl10 degradation

**Figure 7:**
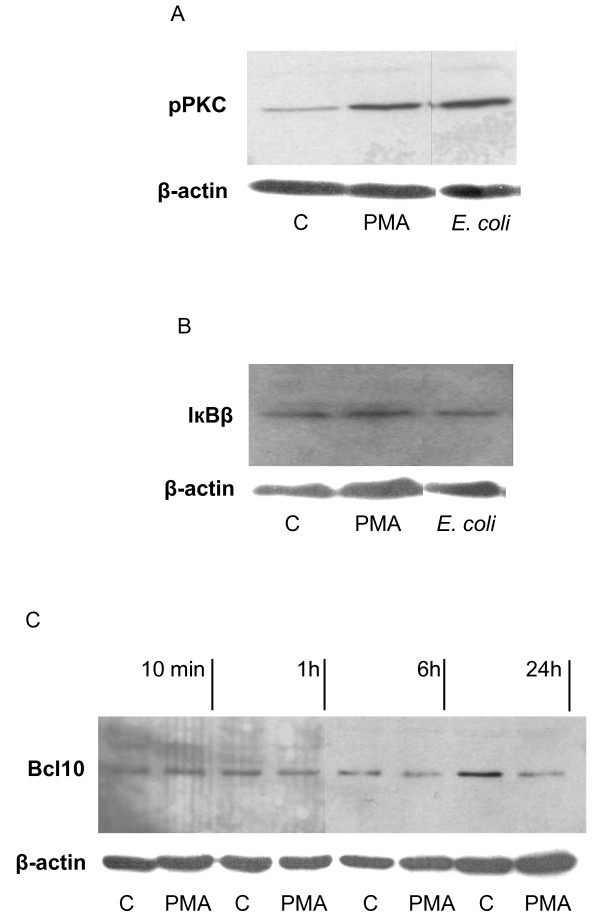
**NF-κB inhibition by PMA correlated to PKC-dependent Bcl10 degradation**. Levels of intracellular protein were assessed following 24 h stimulation with PMA (162 nM) or HK *E. coli *(5 × 10^7 ^CFU/ml). **(A) **Phospho-PKC increased in response to PMA and HK *E. coli *stimulation. **(B) **IκB decreased following stimulation with HK *E. coli *indicating NF-κB activation. **(C) **Bcl10 activation was inhibited following long-term stimulation with PMA, which explains the inhibitory effect of PMA on NF-κB activation. β-actin was used as a loading control. (n = 3).

Western blot analysis revealed an up-regulation of phosphorylated-PKC after 24 h treatment of Jurkat T-cells with PMA or HK *E. coli *(figure 7a), while IκBβ levels remained unaffected (figure 7b). Bcl10 is a signalling protein that acts upstream of NF-κB in concert with CARMA1 and MALT1 and has been suggested to directly regulate NF-κB activity in T-cells [27]. Therefore, Bcl10 activation was evaluated in both control and PMA stimulated cells after 10 min, 1 h, 6 h, and 24 h using western blot analysis. The Bcl10 levels decreased following treatment with PMA, while in control cells, Bcl10 returned to higher levels by 24 h (figure 7c). This suggests that Bcl10 is involved in the PMA dependent inhibition of NF-κB activation.

## Discussion

NF-κB and AP-1 are critical regulators of inflammatory responses, proliferation and differentiation of T-cells [28-30], however, the signal transduction and subsequent cytokine/chemokine expression is not fully understood. The aim of the present study was to investigate IL-6 and CXCL8 regulation by NF-κB and AP-1 in Jurkat T-cells.

Our results demonstrated that PMA induced AP-1 activation, indicating a specific activation of the MAPK pathway. Furthermore, we demonstrated that PMA dependent AP-1 activation in T-cells was delayed (>2 h) and increased following long-term treatment. MAPK is one of the main signalling pathways in T-cells that regulate cell- and transcriptional activation [31,32]. Several studies [20,33,34] have indicated the importance of AP-1 in T-cell activation and the induction of inflammatory responses [35], including pro-inflammatory cytokine release. In contrast to AP-1, NF-κB activity rapidly increased (1 min) during PMA exposure followed by a down-regulation to the lowest levels at 6 h. This is in line with Park and colleagues [36] who demonstrated a rapid increase in NF-κB activity following short-term stimulation with PMA, but prolonged challenge resulted in a persistent inhibition of NF-κB. They showed that the inhibition of NF-κB was due to PKC-dependent degradation of IκB kinase β and γ in response to PMA. Interestingly, the HK *E. coli *exposure induced NF-κB activation without affecting AP-1 activity. Wang and colleagues [37] reported an elevated inflammatory response by obtaining expression of IL-6 following the exposure of T-cells to peptidoglycan. Reduction in NF-κB by calcium ionophore following HK *E. coli *stimulation may be due to the Ca^2+ ^binding protein calmodulin (CaM), which has been shown to negatively regulate c-Rel when activated [38]. The regulation of NF-κB and AP-1 observed in the present study was in agreement with earlier studies.

T-cells produce a broad range of pro- and anti-inflammatory cytokines, including IL-2, IL-6, CXCL8, TNF and IL-10, in response to infections or other stress factors [39]. The assessment of Jurkat T-cell inflammatory responses (Table 1) revealed an enhanced IL-2 expression upon exposure to PMA or HK *E. coli *due to PKC activation [40]. In addition to the transcription factor NF-κB, AP-1 binding sites have been identified in the IL-6 promoter region, indicating multiple regulation [22]. AP-1 and NF-κB have also been demonstrated to regulate CXCL8 expression during induction of inflammatory responses in T-cells [12]. Furthermore, IL-6 and CXCL8 gene-expression is associated with an early immune response in Jurkat T-cells [41]. In the present study, both PMA and HK *E. coli *resulted in comparable increases in IL-6 while PMA was more potent at activating CXCL8 release. PMA and HK *E. coli *treatment also induced TNF expression. TNF is one of the first cytokines induced by T-cells [42] and its expression is regulated by calcineurin, NFAT and ATF-2/Jun [20]. However, PMA-stimulated Jurkat T-cells showed no difference in IL-10 expression indicating an induced inflammatory response. HK *E. coli *treatment resulted in a significant reduction of IL-10 expression. The anti-inflammatory cytokine IL-10 is known to inhibit T-cell activation, proliferation and the expression of pro-inflammatory cytokines, such as IL-2, IL-5 and INF-γ [39,43] and regulate inflammatory responses by inducing T-cell anergy [44].

The time course analysis of cytokine expression showed a correlation between AP-1 and the chemokine CXCL8 where CXCL8 expression was significantly elevated already at 2-6 h after PMA exposure. The CXCL8 expression did not correlate with the early NF-κB activation (1 min) or with the down-regulation of NF-κB at 2 h post exposure. Both IL-6 and TNF expression were up regulated between 6-24 h. During this period, NF-κB increased from its minimum level at 6 h. PKC has been shown to be associated with the activation of AP-1, but not with NF-κB activation of the IL-2 promoter [45]. Mutation of the NF-κB site did not affect IL-2 expression, whereas mutation of the AP-1 site or PKC depletion almost revoked IL-2 release. These observations indicate that the MAPK pathway and the transcription factor AP-1 play an important role in the induction of inflammatory responses in Jurkat T-cells [12,20,22]. The obtained results signify that CXCL8 was primarily regulated through the MAPK pathway.

The NF-κB activation inhibitor (NAI) showed specificity against NF-κB and resulted in a complete IL-6 inhibition following induction with PMA and significant IL-6 reduction after HK *E. coli *treatment. CXCL8 was highly up regulated by PMA and the addition of NAI resulted in a minor reduction in CXCL8 expression. Furthermore, CXCL8 expression was not affected by NAI following HK *E. coli *treatment, indicating a lack of correlation between CXCL8 and NF-κB. Further analysis of CXCL8 expression revealed a down-regulation by PKC- and JNK- inhibitors, suggesting an involvement of AP-1 via PKC and JNK, respectively. Analysis of gene expression further confirmed that *il-6 *and *cxcl8 *were upregulated by PMA. Expression of *il-6 *dropped below basal levels following inhibition of NF-κB and JNK whereas *cxcl8 *remained elevated above basal levels (16.5-fold and 7.1-fold respectively) following the same treatment. Furthermore, inhibition of PKC did not result in a significant decrease of *il-6 *or *cxcl8*, which is in accordance with protein data (figure 6). These results suggest that NF-κB is involved in IL-6 regulation and release while it is not required for the expression of CXCL8 in Jurkat T cells.

The transcription factor NF-κB is responsible for a rapid immune response which is followed by an increase in transcription of IκB thus inhibiting NF-κB [46]. Activation of NF-κB in Jurkat T-cells is dependent on Bcl10 activation, which in turn is regulated by PKC. Recent studies have established the importance of a protein complex consisting of CARMA1, Bcl10 and MALT1 (CBM), in the induction of NF-κB. Investigating Bcl10, Scharschmidt and colleagues [47] demonstrated that it is a critical regulator of NF-κB activity. Down-regulation of Bcl10 from signals transduced via the TCR/CD28 and PKC resulted in a concomitant down-regulation of NF-κB. They suggested that Bcl10 is initially activated by TCR/PKC but that continued activation (>1 h) promotes its degradation. Narayan and colleagues [27] suggested that deletion in any of the three CBM complex proteins impairs antigen-receptor dependent activation of NF-κB. They showed that NF-κB activation via Akt requires CARMA1 and acts in cooperation with PKC following short-term exposure (30 min) of Jurkat T-cells with PMA. Akt phosphorylates and thus activates Bcl10. These studies indicate that PKC is crucial for NF-κB activation following short-term treatment through signals via membrane bound receptors such as the TCR and the co-stimulatory receptor CD28. Thus, the CBM complex proteins play a key role in this signalling process. PMA diffuses into the cytosol and directly activates PKC since it is an analogue to diacylglycerol [48]. Several studies have demonstrated that inhibition of PKC blocks NF-κB and AP-1 activity, suggesting a direct regulation of these transcription factors by PKC [49,50]. PKC is activated at an early stage following T-cell stimulation and is therefore an important regulator of downstream inflammatory signalling pathways leading to cytokine expression [50]. We have shown that phosphorylated-PKC is up regulated in response to PMA and HK *E. coli*, indicating an association between PKC and the transcription factors AP-1 and NF-κB. Bcl10 levels were down-regulated following extended treatment of Jurkat T-cells with PMA. In the control groups, a loss of Bcl10 occurred after 6 h, followed by an increase after 24 h, which is in accordance with the observed NF-κB activity (figure 7). These results are supported by an earlier study [47], demonstrating that prolonged PKC activation by PMA leads to an inhibition of Bcl10 and NF-κB.

It has been shown that NF-κB is an important transcription factor complex involved in almost every aspect of cell regulation including apoptosis, differentiation, proliferation and initiation of immune responses [51-53]. NF-κB is constitutively active in many human malignancies, which makes it an attractive therapeutic target [54]. Elevated CXCL8 levels during chronic inflammation result in an enhanced recruitment of immune cells to the site of infection, which may lead to the development of autoimmune diseases following secretion of pro-inflammatory cytokines. In HIV infected persons, serum CXCL8 levels are elevated and this could recruit more T-cells potentially leading to a more rapid progression of the disease since there will be more T-cells available to infect.

## Conclusion

In the present study, IL-6 release was found to be associated with NF-κB activity while CXCL8 release more closely correlated with AP-1 activity. Treatment of Jurkat T-cells with PMA was more potent than HK *E. coli *at elevating the CXCL8 levels. PMA induced AP-1 activation and down-regulated NF-κB while HK *E. coli *up-regulated NF-κB without affecting AP-1 activity. In addition, the temporal induction pattern of AP-1 correlated to the release of CXCL8 while IL-6 followed the NF-κB activity. Likewise, blocking NF-κB activation resulted in a complete inhibition of IL-6 while the CXCL8 levels remained elevated as shown both at the protein and mRNA level. Furthermore, the CXCL8 release was down-regulated by inhibition of JNK activity. The present study indicates that in Jurkat T-cells, IL-6 is regulated through NF-κB while CXCL8 regulation is independent of NF-κB and closely associated with AP-1 activation.

## Materials and methods

### Chemicals

The following chemicals were used in the present study: PMA (Phorbol 12-myristate 13-acetate, (Sigma #P1585, USA)); NF-κB activation inhibitor (NAI), (InSolution™ NF-κB Activation Inhibitor, Calbiochem #481407, USA); JNK inhibitor, (InSolution™ JNK Inhibitor II, Calbiochem #420128, USA); PKC Inhibitor, (InSolution™ Bisindolylmaleimide I, Calbiochem #203293, USA); Calcium Ionophore, (Calcium Ionophore A23187 mixed calcium magnesium salt, Sigma #C5149, USA).

### Heat killed (HK) Escherichia coli

*E. coli *MG1655 were grown on Luria-Bertani (LB) agar and incubated at 37°C overnight. One colony was inoculated into 10 ml LB broth and incubated on a shaker (200 rpm) at 37°C overnight. The bacteria were centrifuged for 10 min at 3000 × g, washed with 3 ml phosphate buffered saline (PBS; 8 g NaCl, 1.16 g Na_2_HPO_4_, 0.2 g KH_2_PO_4_, 0.2 g KCl, pH7) and resuspended in 50 μl PBS. The bacteria were killed by heating to 70°C for 1 h. To ensure that the bacteria were killed; 10 μl of the heat-killed suspension was spread on a LB plate and incubated overnight at 37°C.

### Cell culturing, transfection and stimulation

Jurkat T-cells (wild type and TCR deficient- TCR^-/-^) were maintained in 90% RPMI 1640 medium (PAA laboratories, Austria) with 1.5 mM L-glutamine (Invitrogen, USA), 10% foetal bovine serum (Invitrogen, USA) and 1% antibiotic-antimycotic (Invitrogen, USA) and incubated in a stable environment of 5% CO_2 _at 37°C.

The cells were centrifuged at 1000 × g for 8 min and resuspended in fresh media to a final cell density of 1.6 × 10^7 ^cells/ml in a 24-well plate. Reporter plasmid (pNFκB-Luc, pAP1 (PMA)-TA-Luc, pNFκB-SEAP), internal control plasmid (pRL) (Promega, USA) and lipofectamine 2000 (Invitrogen, USA) were added to each well at 0.54 μg/well, 0.06 μg/well and 1.5 μl/well, respectively. Initially, the reporter plasmid and pRL were mixed separately with OptiMEM (Gibco, USA). After 5 min of incubation at room temperature, lipofectamine 2000 was added and the mixture was incubated further for 20 min at room temperature. The transfection was allowed to proceed overnight at 37°C, after which, the cells were centrifuged, the media removed and fresh pre-warmed media added. The cells were pre-incubated with NF-κB, JNK and PKC inhibitors and stimulated in 24-well plates with different concentrations of PMA, HK *E. coli *MG1655 and Calcium Ionophore A23187.

The cells were lysed and luciferase activity (NF-κB and AP-1) was measured using the Dual-Luciferase^® ^reporter assay system (Promega, USA) according to the manufacturer's instructions on a TD 20/20 luminometer (Turner Designs, Sunnyvale, CA). Secreted alkaline phosphatase (NFκB-SEAP, figure 4a, b) levels were measured using Great EscAPe™ SEAP Detection Kit (Clontech, USA).

### Multiplex cytokine assay

Quantification of the levels of cytokines IL-2, IL-6, IL-10 and TNF and the chemokine CXCL8 was performed on culture supernatants using multiplexed biomarker immunoassay kits according to manufacturer's instructions (Bio-Rad Laboratories, Hercules, CA). A Bio-Plex™ 200 readout System was used (Bio-Rad), which utilizes Luminex^® ^xMAP™ fluorescent bead-based technology (Luminex Corp., Austin). Levels were automatically calculated from standard curves using Bio-Plex Manager software (v.4.1.1, Bio-Rad).

### Enzyme-linked immunosorbent assay (ELISA)

ELISA was performed on supernatants from challenged Jurkat T-cells to quantify IL-6, CXCL8 and TNF (BD OptEIA Human IL-6 Elisa Set, BD OptEIA Human CXCL8 Elisa Set and BD OptEIA Human TNF Elisa Set, Biosciences, USA) according to the manufacturer's instructions. Briefly, Jurkat T-cells were stimulated with PMA (162 nM) for 1 h, centrifuged (1000 × g, 8 min) and the supernatants were collected and stored at -80°C until use. Following centrifugation, the cells were resuspended in 1 h aged media, where cells have been grown in, containing PMA. The same procedure was performed to collect media after 2 h and 6 h. The final collection of media was performed after 24 h.

### Western blot analysis

Following stimulation, Jurkat T-cells were centrifuged at 1000 × g for 8 min and lysed on ice for 2 h using sodium hydroxide with the addition of a protease inhibitor cocktail (Roche, Mannheim). The cells were further centrifuged at 8000 × g, 4°C for 10 min and the supernatants were transferred to new tubes. Cytoplasmic proteins (~8 μg) were separated by SDS-PAGE (10%) followed by western blotting using anti-IκBβ, phospho-PKC (pan)(ζ Thr410)(190D10), anti-Bcl10 (Cell Signalling Technology, Boston) and beta-actin (Abcam, Cambridge). Detection was performed following incubation with ECL™ Anti-rabbit IgG, horseradish peroxidase linked whole antibodies (Amersham Biosciences, Buckinghamshire) and developed using ECL™ Western Blotting Detection Reagents (GE Healthcare, UK).

### RNA extraction

Jurkat T-cells were treated with NF-κB, JNK and PKC inhibitors for 2 h in 6-well plates followed by stimulation with 162 nM PMA for 24 h. At sampling the cells were pelleted followed by RNA extraction using 100 μl TRI-reagent (Sigma, USA). This was followed by addition of 100 μl chloroform/isoamylalcohol (24/1). The solutions were mixed by vortexing followed by centrifugation at 12,000 rpm for 15 min at 4°C. The upper phase was transferred to a new tube followed by addition of 100 μl iso-propanol and incubated at room temperature for 10 min. RNA was then pelleted by centrifugation at 12,000 rpm for 15 min at 4°C and washed with 70% ethanol. The RNA pellet was dissolved in 25 μl RNase free water and the yield and ratio (A_260_/A_280_) was determined using NanoVue (GE Healthcare, UK). The samples were stored at -80°C until further use.

### Reverse transcription quantitative PCR (RT-qPCR)

RT-qPCR was used to determine gene expression levels of *il-6 *and *cxcl8 *in response to PMA following inhibition of NF-κB, JNK and PKC. The following primer sequences were used, *il-6*: forward- TGTGAAAGCAGCAAAGAGGCACTG, reverse- ACAGCTCTGGCTTGTTCCTCACTA; *cxcl8*: forward- ACCACACTGCGCCAACACAGAAAT, reverse- AAACTTCTCCACAACCCTCTGCAC. Thermocycling conditions for CYBR Green (Quanta, USA) consisted of a denaturation step for 10 min at 95 °C followed by 60 cycles of 95°C for 1s and 60°C for 30s. Gene expression was analysed using Stratagene (Mx3000p™) (AH diagnostics). The obtained Ct values were normalized against 18S. Initially, all measured 18S Ct values were used to calculate a mean Ct value that was used to determine the ΔCt values for each sample. Gene expression patterns for *il-6 *and *cxcl8 *were then normalized with regard to the samples 18S ΔCt.

### Statistical analysis

Statistical significant differences were determined using two-tailed Student's *t*-test (*p < 0.05; **p < 0.01; ***p < 0.001).

## Abbreviations

PKC: protein kinase C; TCR: T cell receptor; CARMA1: caspase recruitment domain-containing membrane-associated guanylate kinase protein-1; Bcl10: B-cell chronic lymphocytic leukemia/lymphoma 10; MALT1: mucosa associated lymphoid tissue lymphoma translocation gene 1.

## Authors' contributions

HK participated in the design of the study and conducted the lab work. JJ participated in the design of the study and the multiplex assays. PEO coordinated the study and participated in the design of the study. All authors participated in writing, reading and approving the final manuscript.
